# Vitamin D Induction of the Human Antimicrobial Peptide Cathelicidin in the Urinary Bladder

**DOI:** 10.1371/journal.pone.0015580

**Published:** 2010-12-14

**Authors:** Olof Hertting, Åsa Holm, Petra Lüthje, Hanna Brauner, Robert Dyrdak, Aino Fianu Jonasson, Peter Wiklund, Milan Chromek, Annelie Brauner

**Affiliations:** 1 Department of Microbiology, Tumor and Cell Biology, Division of Clinical Microbiology, Karolinska Institutet and Karolinska University Hospital, Stockholm, Sweden; 2 Astrid Lindgrens Childrens Hospital, Karolinska University Hospital, Stockholm, Sweden; 3 Department of Microbiology, Tumor and Cell Biology, Karolinska Institutet, Stockholm, Sweden; 4 Department of Clinical Science, Unit of Obstetrics and Gynecology, Karolinska Institutet and Karolinska University Hospital, Stockholm, Sweden; 5 Department of Urology, Division of Surgery, Karolinska Institutet and Karolinska University Hospital, Stockholm, Sweden; Charité-University Medicine Berlin, Germany

## Abstract

The urinary tract is frequently being exposed to potential pathogens and rapid defence mechanisms are therefore needed. Cathelicidin, a human antimicrobial peptide is expressed and secreted by bladder epithelial cells and protects the urinary tract from infection. Here we show that vitamin D can induce cathelicidin in the urinary bladder. We analyzed bladder tissue from postmenopausal women for expression of cathelicidin, before and after a three-month period of supplementation with 25-hydroxyvitamin D_3_ (25D_3_). Cell culture experiments were performed to elucidate the mechanisms for cathelicidin induction. We observed that, vitamin D *per se* did not up-regulate cathelicidin in serum or in bladder tissue of the women in this study. However, when the bladder biopsies were infected with uropathogenic *E. coli* (UPEC), a significant increase in cathelicidin expression was observed after 25D_3_ supplementation. This observation was confirmed in human bladder cell lines, even though here, cathelicidin induction occurred irrespectively of infection. Vitamin D treated bladder cells exerted an increased antibacterial effect against UPEC and colocalization to cathelicidin indicated the relevance of this peptide. In the light of the rapidly growing problem of resistance to common urinary tract antibiotics, we suggest that vitamin D may be a potential complement in the prevention of UTI.

## Introduction

Urinary tract infection (UTI) remains an important disease and becomes more frequent in women after menopause. Due to the proximity of the highly colonized perineum, the urinary tract epithelium must be able to sense pathogens and elicit a fast innate immune response in order to keep its integrity [1], [2]. Previously we demonstrated that the human antimicrobial peptide cathelicidin was up-regulated upon *E. coli* infection and that it significantly contributes to the protection of the urinary tract in humans and in mice [3]. Here, we investigate vitamin D-induced boosting of cathelicidin in the urinary bladder.

Vitamin D, apart from its well-known task to regulate calcium metabolism, has been recognized to influence innate and acquired immune reactions [4]. Skin exposure to sunlight and dietary intake of fatty fish and dairy products are the predominant sources of vitamin D [5]. In the liver, hydroxylation takes place and 25-hydroxyvitamin D_3_ (25D_3_) is produced. The inactive form 25D_3_ is converted in the kidney to its active metabolite 1,25D_3_ by the hydroxylase CYP27B1 [6]. 1,25D_3_ binds to the vitamin D receptor (VDR) and is transported into the nucleus where it acts as a transcription factor. Recently, it has been shown that also non-renal tissue can convert 25D_3_ to 1,25D_3_ [4]. Several studies have concluded an intimate relationship between vitamin D and cathelicidin production. Vitamin D can induce the human cathelicidin gene (*CAMP*) expression by binding to the vitamin D responsive element (VDRE) of the *CAMP* promoter [7], [8] in various tissues, e.g., keratinocytes [9], respiratory epithelium [10] and monocytes [11]. Consequently, increased synthesis of cathelicidin after vitamin D treatment has been observed.

We hypothesized that vitamin D could influence cathelicidin production also in the urinary tract and thereby help protecting from invading microbes. We show that urinary bladder cells enhance cathelicidin production in response to vitamin D. Moreover, we demonstrate the relevance of these findings in bladder tissue from postmenopausal women receiving 25D_3_ supplementation. We also provide evidence that this increased production has an antimicrobial effect. Our findings indicate that 25D_3_ may be a potential complement in the prevention of UTI.

## Results

### Postmenopausal women have low levels of serum vitamin D which increase after 25D_3_ supplementation

**Figure 1:**
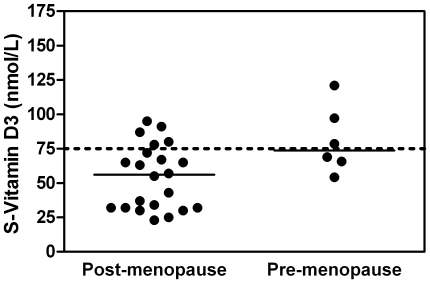
Low levels of vitamin D_3_ in postmenopausal women. Serum vitamin D_3_ levels in postmenopausal women are low compared to premenopausal women, *P*<0.05 (Mann-Whitney test). Individual data are presented with median levels (56 nmol/L and 74 nmol/L). The recommended level of 75 nmol/L is marked with a dotted line.

**Figure 2:**
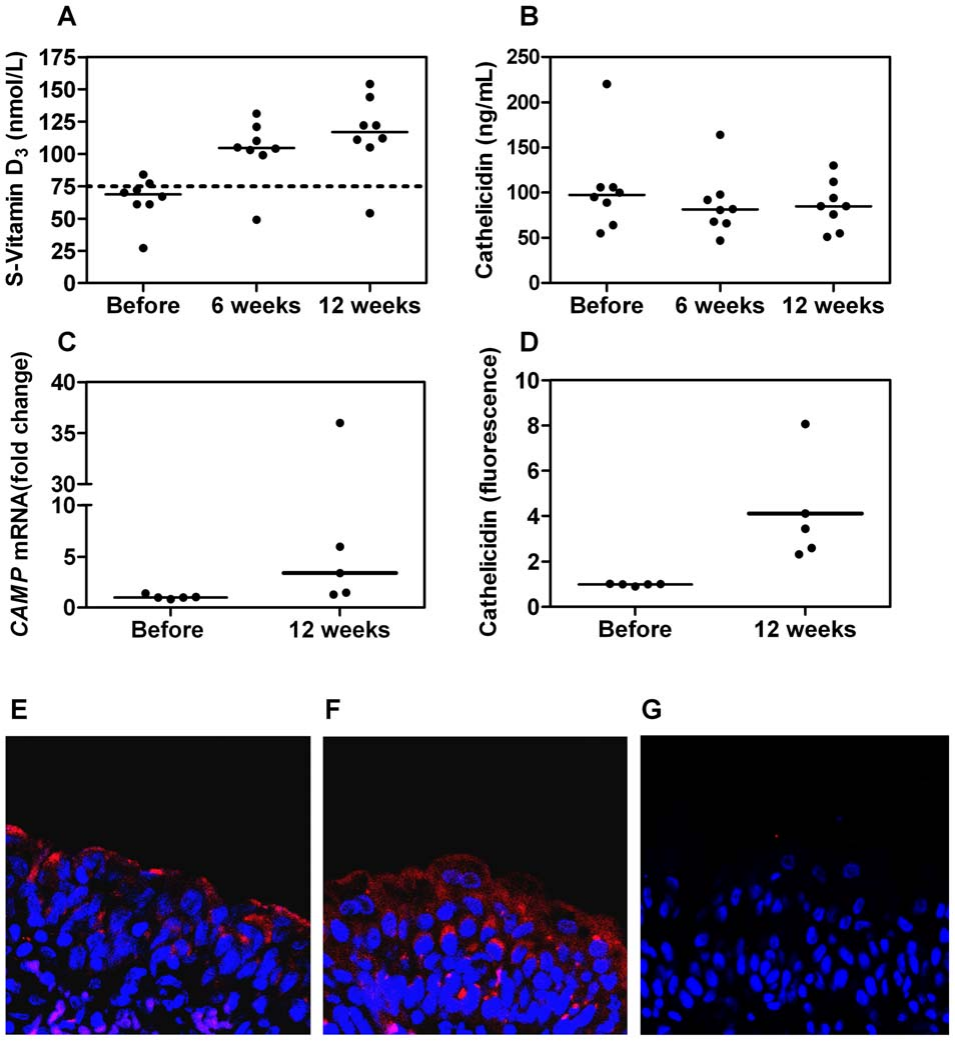
25D_3_ and cathelicidin in serum and bladder tissue of women during a three-month period of 25D_3_ supplementation. Postmenopausal women were treated with 2000 units of oral 25D_3_ daily for 12 weeks. Serum samples were collected before, at 6 and 12 weeks. Serum 25D_3_ levels increased from a baseline median value of 68.5 nmol/L to 104.5 nmol/L at 6 weeks, *P*<0.001 (Wilcoxon rank test), and 117 nmol/L at 12 weeks, *P*<0.01 (Wilcoxon rank test). The dotted line denotes the recommended serum 25D_3_ level of 75 nmol/L and median values are indicated (**A**). Despite treatment with 25D_3_, serum cathelicidin levels did not increase (**B**). *E. coli*-infected bladder biopsies from women receiving 25D_3_ supplementation responded with higher expression of *CAMP* mRNA, *P*<0.05 (Mann-Whitney test) (**C**) and cathelicidin protein, *P* <0.05 (Mann-Whitney test) (**D**) compared to infected pieces prior to supplementation. Fluorescence immunohistochemistry showed increased cathelicidin protein expression in infected bladder biopsies. Tissue samples shown are from the same patient before (**E**) and after (**F**) 12 weeks of 25D_3_ treatment. Cathelicidin is localized in the uroepithelium including the larger umbrella cells closest to the bladder lumen. Staining without primary antibody serves as negative control (**G**). Images are captured at ×63 magnification and depict an area corresponding to 119×119 µm. Cathelicidin is stained with AlexaFluor 594 (red), the cell nucleus with DAPI (blue).

After menopause, women have an increased risk of UTI, coinciding with decreased vitamin D and estrogen levels. We analysed 25D_3_ levels in serum from 22 Swedish healthy postmenopausal and, for comparison, six premenopausal women. The median serum 25D_3_ values were 56 nmol/L and 74 nmol/L, respectively (*P*<0.05). In the group of postmenopausal women, only five women (23%) had 25D_3_ levels above the recommended level of 75 nmol/L [12]. One was vitamin D deficient (<25 nmol/L), nine were insufficient (<50 nmol/L) and the levels of the remaining seven women were between 50 and 75 nmol/L (Figure 1). Eight healthy postmenopausal women were later recruited for vitamin D supplementation, with bladder biopsies taken before and after the treatment period. Intake of 2000 units of 25D_3_ daily increased their median values of 25D_3_ in serum from 68.5 nmol/L to 104.5 nmol/L after 6 weeks (*P*<0.001) and further to 117 nmol/L after 12 weeks (*P*<0.01, Figure 2A). Notably, no correlation was seen between serum levels of 25D_3_ and cathelicidin in the serum before and after 6 or 12 weeks treatment (Figure 2B). Calcium, phosphate and parathyroid hormone were within the physiological range and not affected by 25D_3_ treatment.

### 25D_3_ increases CAMP expression in *E. coli-*infected urinary bladder epithelium

Vitamin D has been shown to activate the antimicrobial peptide cathelicidin in a range of human cell types [13]. Therefore, we investigated if this process also occurs in the urinary bladder. Vitamin D treatment *per se* did not increase cathelicidin mRNA or protein in bladder tissue, as determined by quantitative RT-PCR and microscopy, respectively (data not shown). Bladder biopsies taken before and after supplementation were also infected with *E. coli* CFT073 *ex vivo*. After 25D_3_ supplementation, we found an increase in *CAMP* expression upon infection compared to biopsies prior to supplementation (*P*<0.05, Figure 2C). Likewise, an increase in cathelicidin protein expression was observed (*P*<0.05, Figure 1D). Confocal microscope images showed that before 25D_3_ intake, infection-induced cathelicidin production was restricted mainly to the superficial umbrella cells (Figure 1E). After supplementation however, more cathelicidin was produced and was also seen in cells located deeper in the bladder epithelium (Figure 2F).

### The converting enzyme CYP27B1 is present in the human urinary bladder

**Figure 3:**
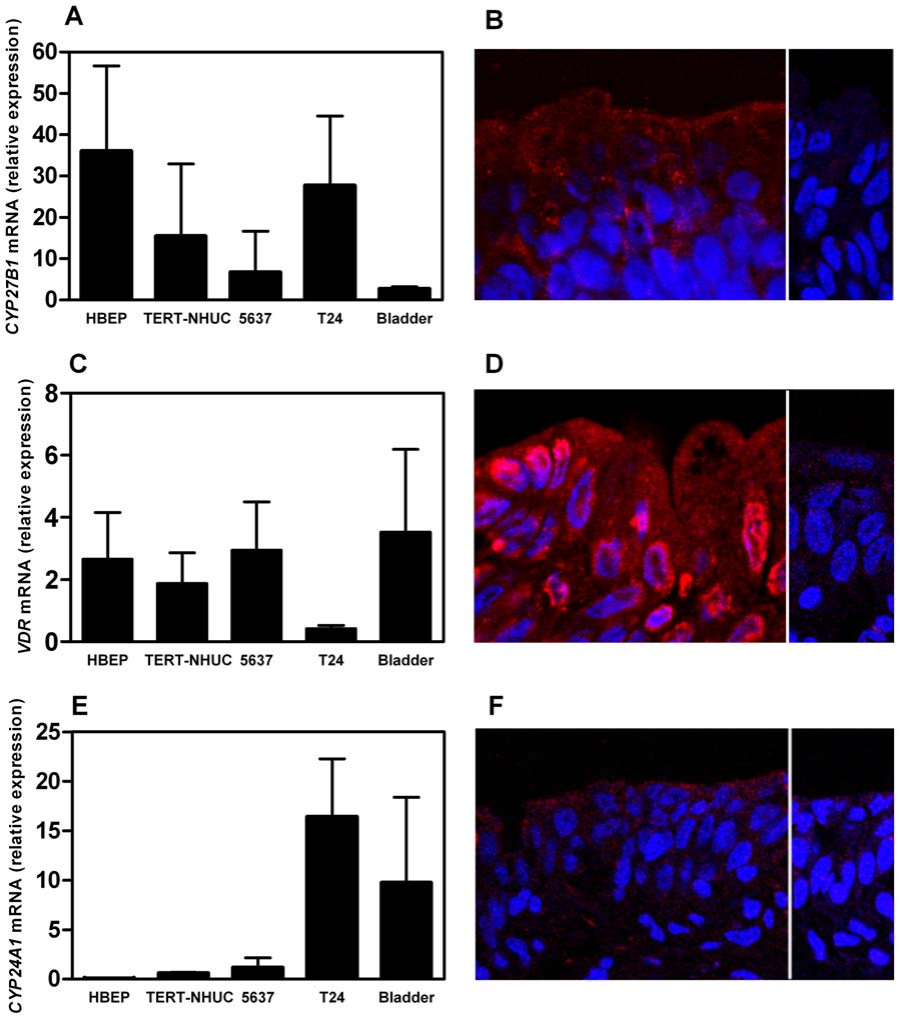
Bladder epithelial cells are equipped with the machinery for local 25D_3_ conversion and activity. Expression of CYP27B1, VDR and CYP24A1 was detected on mRNA level in normal bladder cells (HBEP) and three bladder cells lines (TERT-NHUC, 5637 and T24) by RT-PCR (**A**, **C**, **E**) and on protein level in tissue from bladder biopsies by fluorescence immunohistochemistry (**B**, **D**, **F**). mRNA expression results were normalized to expression in the kidney epithelial cell line A498. The proteins are stained with AlexaFluor 594 (red), the cell nucleus with DAPI (blue). CYP27B1 protein was detected in the bladder mucosa where it was evenly distributed in the bladder epithelial layers with an abundance of staining seen in the peri-nuclear area, consistent with its association to mitochondria (**B**). VDR protein was localized in the cytoplasm and nucleus (**D**). Low levels of the CYP24A1 protein were detected in bladder mucosa (**F**). All images are captured at ×63 magnification and the right panels of images B, D and F show controls stained without primary antibodies against CYP27B1, VDR and CYP24A1, respectively.

To explain the effect of 25D_3_ on cathelicidin production in the bladder of vitamin D-supplemented women, we hypothesized that conversion of 25D_3_ to 1,25D_3_ takes place in the bladder epithelium. Therefore we investigated whether CYP27B1, the enzyme necessary for this conversion, is present in bladder epithelial cells. Using RT-PCR, we found *CYP27B1* mRNA in normal human bladder cells and tissue, and in all three bladder cell lines studied (Figure 3A). By immunofluorescence microscopy, we could detect the presence of CYP27B1 in bladder tissue sections (Figure 3B), localized throughout the cytoplasm with an abundance of staining seen in the peri-nuclear area, consistent with its association to mitochondria [14].

### Inactive 25D_3_ is converted to active 1,25D_3_ in bladder epithelial cells

Based on the findings that bladder epithelial cells are equipped with the machinery for local 25D_3_ conversion, we investigated the biological function of CYP27B1 in bladder epithelial cells. Normal human bladder cells were exposed to 25D_3_ for 24 hours. Conversion to active vitamin D was confirmed by the presence of 1,25D_3_ in the cell culture supernatant at a concentration of >0.4×10^−9^ M. The concentration of 1,25D_3_ in culture medium from untreated cells and 25D_3_ supplemented medium without cells was below detection limit (<0.02×10^−9^ M).

### The vitamin D receptor is expressed in the urinary bladder and in bladder epithelial cells

To up-regulate cathelicidin, active 1,25D_3_ binds to the VDR. Thus, we investigated the presence of VDR in bladder epithelial cells and bladder tissue from biopsies. Bladder cells and bladder tissue expressed *VDR* mRNA constitutively, most abundantly in normal human bladder cells and in cell line 5637 (Figure 3C). VDR protein was detected in bladder epithelium by immunofluorescence microscopy. On a cellular level, it was distributed both in the cytoplasm and in the nucleus (Figure 3D).

### The 1,25D_3_ metabolizing enzyme CYP24A1 is expressed in the urinary bladder and in bladder epithelial cells

Since the 25-hydroxyvitamin D_3_ 24-hydroxylase (CYP24A1) is considered important in determining the biological half-life of 1,25D_3_ [15], we examined the expression of this enzyme in the urinary bladder tissue and in the cell lines used in this study. Low levels of gene-specific mRNA were detected in all cell lines except for T24 cells, which expressed *CYP24A1* at higher levels (Figure 3E). Bladder tissue showed both mRNA and detectable but low protein expression of CYP24A1 (Figure 3F).

### 1,25D_3_ and 25D_3_ enhance cathelicidin production

**Figure 4:**
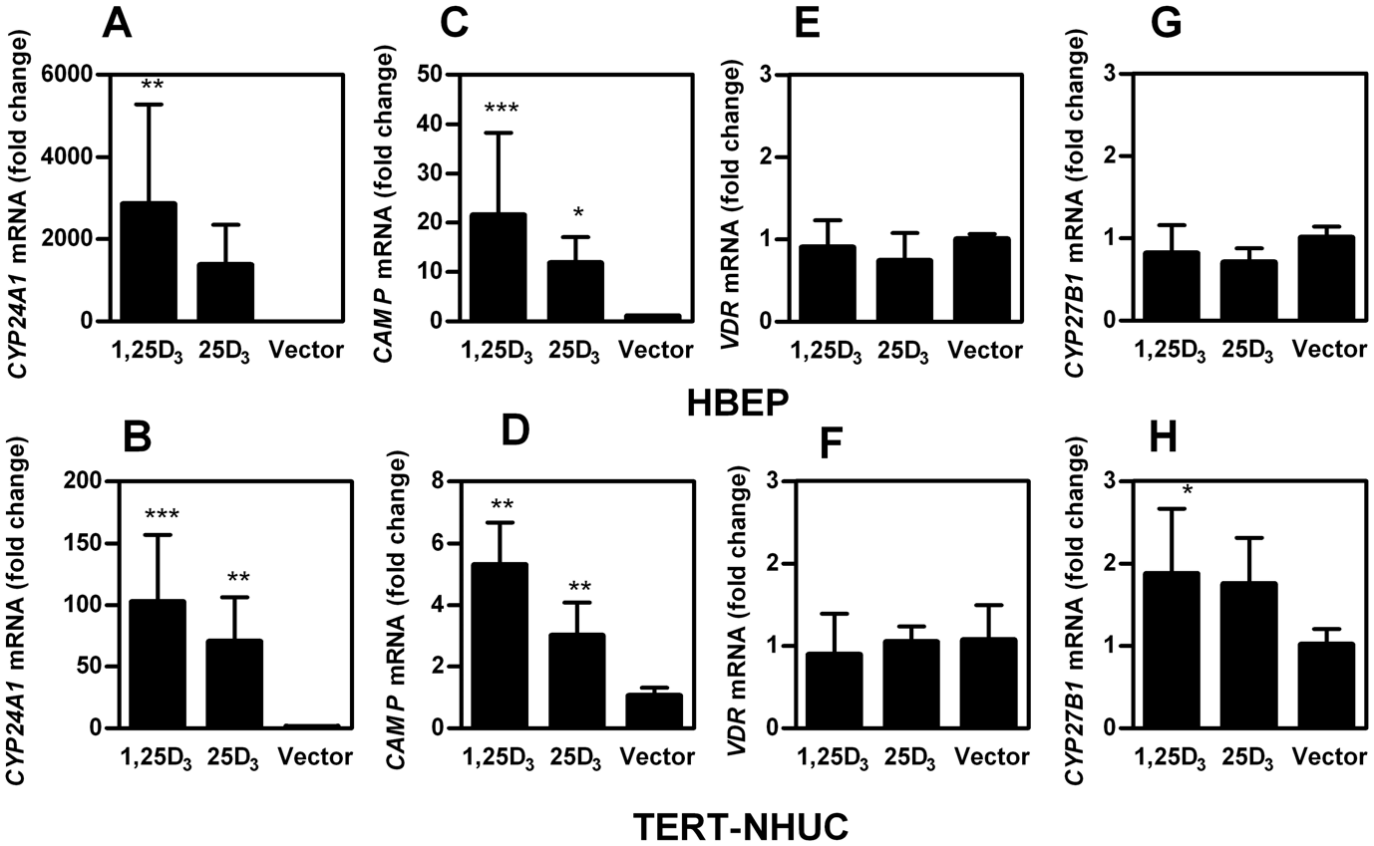
Human bladder cells respond with *CYP24A1* and *CAMP* induction to 1,25D_3_ and 25D_3_. Normal bladder cells (HBEP, upper panel) and TERT-NHUC bladder cells (lower panel) were stimulated with 10^−7^ M 25D_3_ or 10^−8^ M 1,25D_3_ for 24 hours. Levels of gene-specific mRNA for *CYP24A1* (**A**, **B**), *CAMP* (**C**, **D**), *VDR* (**E**, **F**) and *CYP27B1* (**G**, **H**) in stimulated cells were determined and are presented as fold change compared to vector-treated control cells. Data presented are pooled from three independent experiments and shown as mean and standard deviation; ***P<0.001, **P<0.01, *P<0.05 (ANOVA with Dunnett's post-test).

We further investigated the biological effect of 25D_3_ and 1,25D_3_ on vitamin D-dependent gene expression in bladder cells. As a negative feedback regulator in vitamin D metabolism, the hydroxylase CYP24A1 is strongly induced by 1,25D_3_ [15]. In response to 1,25D_3_, expression of *CYP24A1* was highly up-regulated in normal bladder cells and TERT-NHUC (Figure 4A and B). *CYP24A1* up-regulation was also observed after incubation with 25D_3_, indicating conversion of 25D_3_ to 1,25D_3_ (Figure 4A and B). An up-regulation of *CAMP* in the presence of 1,25D_3_ or 25D_3_ was observed in all cell lines tested. Normal bladder cells showed higher induction than TERT-NHUC bladder cells, 21-fold and 5-fold respectively (Figure 4C and D). In the bladder epithelial cell lines T24 and 5637, 1,25D_3_-mediated *CAMP* induction was 16-fold and 140-fold, respectively (data not shown). The expression of *VDR* was not influenced by 1,25D_3_ or 25D_3_ (Figure 4E–F). A small induction of *CYP27B1* was observed in TERT-NHUC bladder cells (Figure 4G).

**Figure 5:**
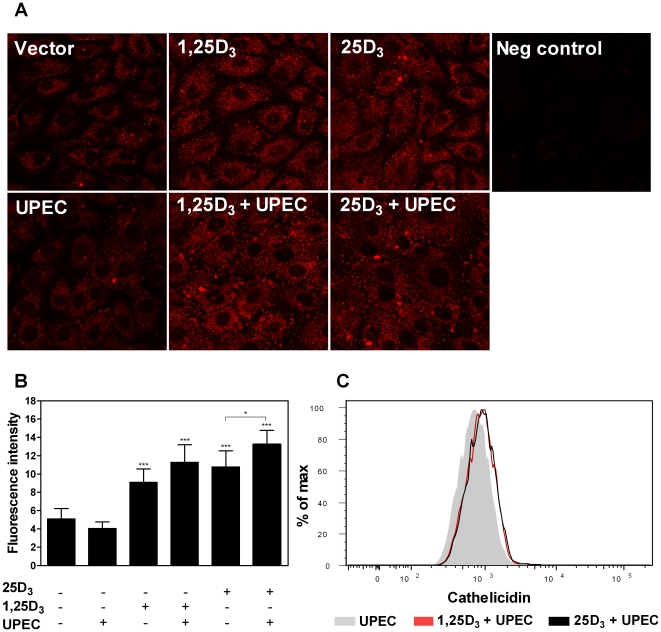
1,25D_3_ or 25D_3_ in combination with *E. coli* increased cathelicidin protein in bladder epithelial cells. Production of cathelicidin increased in TERT-NHUC bladder cells after treatment with 1,25D_3_ or 25D_3_, and was further increased by infection with *E. coli* CFT073 (UPEC). Images are captured at ×63 magnification and depict an area corresponding to 119×119 µm (**A**). The control image (Neg control) is shown in the right upper panel. Fluorescence intensity was quantified and is presented as mean values with standard deviation; ***P<0.001, *P<0.05 (ANOVA with Dunnett's post-test) (**B**). An increase in cathelicidin protein was also recorded by flow cytometry in *E. coli*-infected TERT-NHUC bladder cells treated with 1,25D_3_ or 25D_3_. Grey is vector +*E. coli*, red is 1,25D_3_ 10^−8^ M+*E. coli* and black is 25D_3_ 10^−7^ M+*E. coli* (**C**).

To determine whether *CAMP* gene expression correlates with increased protein production, immunofluorescence microscopy and flow cytometry was performed. Cathelicidin levels increased significantly in TERT-NHUC cells after 48 hours of 1,25D_3_ or 25D_3_ treatment (*P*<0.001, Figure 5A and B). Cells pre-treated with vitamin D and infected with *E. coli* CFT073 expressed even higher levels of cathelicidin (*P*<0.05 for 25D_3_ +/− UPEC, Figure 5A and B). Flow cytometry confirmed an increase of cathelicidin in 25D_3_ and 1,25D_3_ treated cells after *E. coli* infection (Figure 5C).

### Vitamin D promotes cell-mediated killing of UPEC

**Figure 6:**
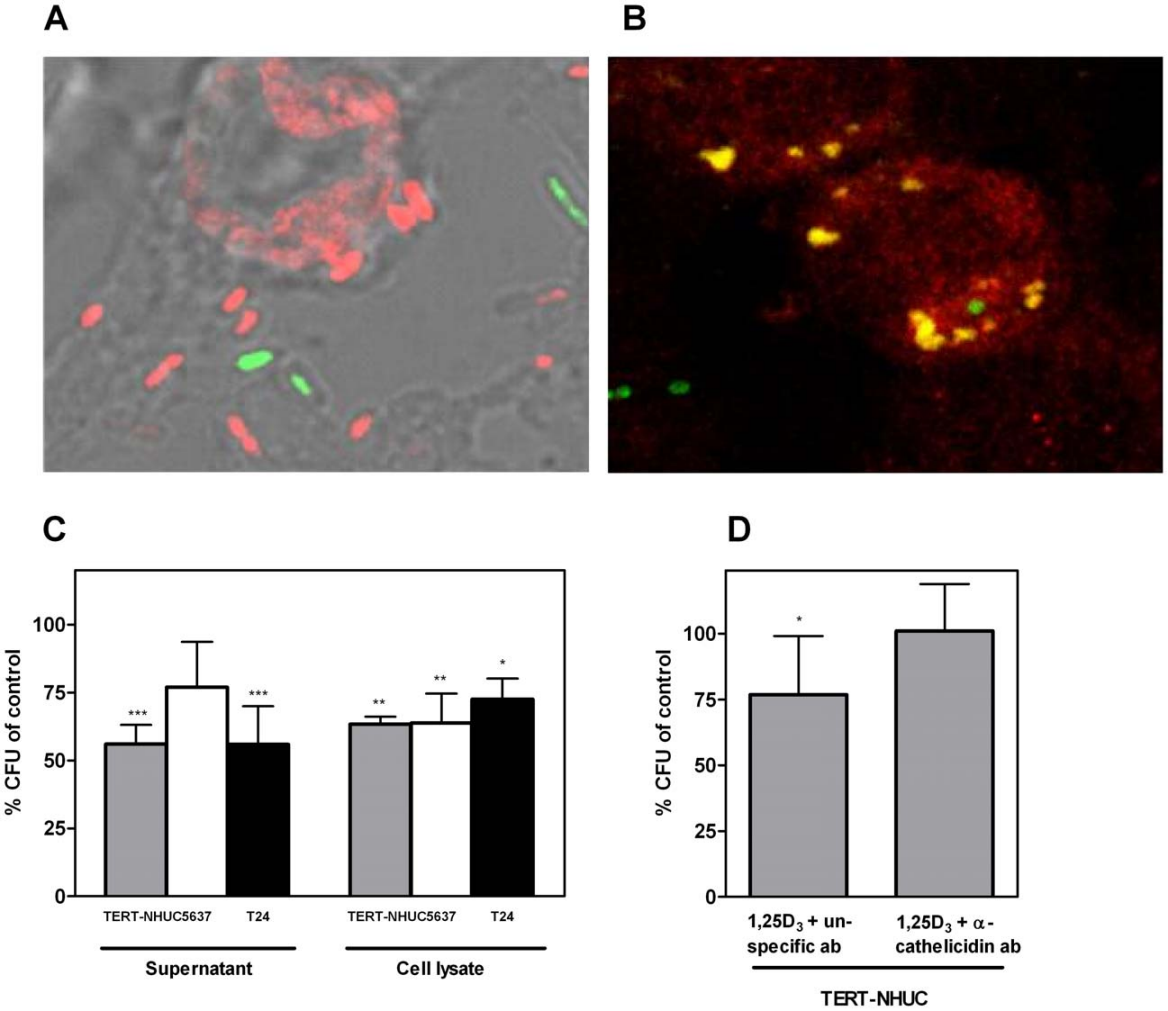
Vitamin D increases the cathelicidin-mediated antibacterial effect of bladder epithelial cells. Cell-associated UPEC are killed as determined with Live/Dead staining where live bacteria are stained green and dead bacteria are stained red (**A**). UPEC attach and invade bladder cells and are colocalized with cathelicidin. GFP-labelled *E. coli* CFT073 is green and cathelicidin is stained with AlexaFluor 594 (red); regions of colocalization are yellow (**B**). The antibacterial effect induced by 1,25D_3_ was measured in medium from TERT-NHUC, 5637 and T24 bladder cells after stimulation for 48 hours. *E. coli* CFT073 was incubated in conditioned medium from treated cells for 90 minutes (Supernatant). Cells from the same experiment were harvested and lysed (Cell lysate). The resulting bacterial count is shown as percentage of vector treated control cells. Mean values with standard deviation are shown from three independent experiments; ***P<0.001, **P<0.01 and *P<0.05 (ANOVA with Dunnett's post-test) (**C**). Adding a neutralizing anti-cathelicidin antibody (α-cathelicidin ab) to the conditioned medium from TERT-NHUC bladder cells blocked the antibacterial effect, whereas conditioned medium in the presence of an irrelevant antibody maintained its antibacterial activity. Mean values with standard deviation are shown from four independent experiments; *P<0.05 (ANOVA with Dunnett's post-test) (**D**).

Next, we investigated the biological effect of the increased cathelicidin production in vitamin D-treated cells. We therefore assessed the antibacterial properties of treated *versus* untreated cells against UPEC strain CFT073. By confocal microscopy, we visualized antibacterial activity on a cellular level. Killing of cell-associated bacteria was demonstrated by Live/Dead staining (Figure 6A). The significance of cathelicidin was demonstrated by colocalization of cathelicidin with bacterial cells (Figure 6B). To quantify antibacterial activity and the contribution of cathelicidin, antibacterial assays in conditioned medium were performed. Supernatant from bladder epithelial cells treated with 1,25D_3_ decreased growth of *E. coli* CFT073 by 44% in TERT-NHUC, 47% in T24, and by 23% in 5637 cells. Likewise, growth in cell lysates was inhibited by 36% in 5637 and TERT-NHUC cells and by 27% in T24 cells (Figure 6C). 1,25D_3_ in medium without cells had no antibacterial effect (data not shown). In the presence of a cathelicidin-neutralizing antibody, the antimicrobial effect of the cell supernatant was significantly reduced, confirming the impact of cathelicidin (Figure 6D).

## Discussion

Here we show that oral 25D_3_ supplementation of healthy postmenopausal women prepares the bladder tissue to fight *E. coli* infection by increased production of cathelicidin upon bacterial contact. 25D_3_ is locally converted to 1,25D_3_ in bladder epithelial cells, binds to VDR, which leads to up-regulation of *CAMP* and synthesis of cathelicidin. The increased production in turn enhances the direct antibacterial effect on UPEC.

Women in menopause are prone to UTI. This is traditionally mainly attributed to anatomical reasons and mucosal atrophy. With age, VDR expression decreases [16] and there are reports suggesting that women have lower vitamin D intake than men [17]. The most important source of vitamin D is UVB-induced photoactivation of previtamin D in the skin [18]. Because of its geographical latitude, Sweden has only few hours of sunlight during winter season. Hence, low levels of vitamin D in the study population were anticipated. Still, it was surprising that 77% of otherwise healthy postmenopausal women did not have sufficient vitamin D levels. We therefore hypothesized that vitamin D may be involved also in the defence against UTI, and that this is mediated by cathelicidin.

The relatively high dose of 25D_3_ used in this study did not affect constitutive cathelicidin production in the urinary bladder. However, a local up-regulation was observed in the tissue in response to infection with UPEC. Interestingly, 2000 units of 25D_3_ daily, about five times the recommended daily dose [19], showed no increase in serum cathelidicin and did not influence serum calcium levels. This is in line with previous reports showing no correlation between high doses of vitamin D_2_ and circulating levels of cathelicidin [20]. Thus, we speculate that 25D_3_ prepares the cells to produce more cathelicidin only when needed to protect the urinary tract from bacterial infection.

Vitamin D is a well-studied compound and currently supplementation of 25D_3_ is being widely recommended [12]. Clinical studies using 10 times the dose given in the present study reported serum concentrations of 400 nmol/L with no hypercalcemia or hypercalcuria [21]. The risk for unwanted side effects with the given dose in our study is therefore limited. Vitamin D has successfully been used to enhance the immunity to pathogens in the respiratory tract [22], [23]. For this local protection to occur, conversion of 25D_3_ to active 1,25D_3_ in peripheral tissue is needed. Here, we localized the converting enzyme CYP27B1 in the uroepithelium and in normal bladder cells. Activity of CYP27B1 was indicated by the detection of 1,25D_3_ in culture medium from 25D_3_ treated bladder cells, suggesting a local function of 1,25D_3_ within the bladder mucosa. We also confirm earlier studies showing VDR expression in bladder tissue [24]. Normal bladder cells and 5637 had the highest induction of *CAMP*, coinciding with higher VDR levels. This raises the question if age-dependent loss of VDR may lead to increased risk of UTI. The results from cell lines differed from bladder tissue with respect to CYP27B1 and CYP24A1. This may well reflect the difference between a monolayer of cells in culture and the complex nature of the multilayered urothelium with cells at different level of differentiation.

Both 25D_3_ and 1,25D_3_ up-regulated cathelicidin production in various bladder cell lines, and as a result, treated cells mediated increased antibacterial activity. In the investigated cells, induction was observed by vitamin D without additional stimuli. This is in line with what previously has been demonstrated in respiratory epithelial cells [25]. In contrast, in monocytes additional stimulation via Toll-like receptor (TLR) is needed to induce cathelicidin production [26]. We cannot rule out that our observed differences in tissue *versus* cultured cells are due to lower concentrations of 25D_3_ and 1,25D_3_ in the target cells *in vivo*. It is plausible that, at these concentrations, additional stimuli such as TLR signalling are needed to induce detectable cathelicidin levels. On the other hand, bacteria alone did not increase cathelicidin intracellularly under the experimental conditions used here. This has previously been observed for non-pathogenic *E. coli* and *Neisseria*; in contrast, even a down-regulation was observed [27]. We believe that the presence of vitamin D is sufficient and necessary to induce cathelicidin, and might further be enhanced by the presence of bacteria.

Our results suggest a role for vitamin D in the protection against UTI. So far, no data are available on vitamin D levels and UTI incidence. Still, there is an established link between vitamin D deficiency and tuberculosis [28], [29] and viral respiratory infections [30], [31]. Accordingly, low levels of VDR and VDR polymorphisms have been associated with such infections in young children [32].

In light of the emerging resistance to antibiotics used against UTI [33], new treatment strategies are needed. Our data suggest that vitamin D can stimulate an increased production of the antimicrobial peptide cathelicidin. By inducing and activating cathelicidin with vitamin D, a local rather than a systemic effect can be achieved. This could offer selective and site-specific treatment of pathogens without perturbing commensal microbes elsewhere in the body. Likewise, systemic effects of 25D_3_ treatment secondary to cathelicidin induction seem unlikely. This could make 25D_3_ an effective and safe way of activating the endogenous antimicrobial response locally at the site of infection. Determining the vitamin D status of individuals with a history of UTI may be of importance to evaluate their ability to fend off intruding bacteria. Supplementation to restore proper vitamin D levels may therefore help preparing the bladder epithelium to mount a stronger and faster immune response once bacteria enter the bladder.

## Materials and Methods

### Human participants

The study was approved by Regionala etikprövningsnämnden (EPN), Stockhom (The Regional Ethics Committé, Stockholm), Protocol 2008/968-31. It was performed in accordance to the Helsinki Declaration. Informed written consent was obtained from volunteers involved in the study. Initially, 22 postmenopausal and six premenopausal Swedish women of European descent (median age 59) were analyzed regarding their serum vitamin D levels. Eight additional women (median age 62) were later recruited to receive daily oral supplementation with 2000 units of 25D_3_ (Recip, Meda Pharmaceuticals) for 12 weeks. They had no history of urinary tract abnormalities and none of the women received any dietary supplements or hormonal treatment at the time of the study. Before starting vitamin D supplementation, cystoscopy was performed and superficial biopsies were taken from the urinary bladder wall. After 12 weeks, follow-up biopsies were taken the same way. Out of these eight women, five women underwent the complete procedure. Serum samples were collected before as well as 6 and 12 weeks after treatment and analyzed for 25D_3_, cathelicidin, calcium, glucose, parathyroid hormone and phosphate. Urinalysis dipsticks (Nitristic, Raerad Products) were used to rule out bacteriuria.

### Measurement of 25D_3_ and 1,25D_3_

Serum samples were protected from light and stored at −80°C prior to analysis. Total 25D_3_ analyses were performed using LIASION 25-OH Vitamin D TOTAL kit, an antibody-based chemiluminescence assay, detection limit 10 nmol/L (DiaSorin Inc.). 1,25D_3_ in cell supernatants was determined with the Gamma-B 1,25-dihydroxyvitamin D RIA kit, detection limit 0.02 nmol/L, cross-reactivity between 25D_3_ and 1,25D_3_ 0.001% (Immunodiagnostic Systems).

### Measurement of serum cathelicidin

Cathelicidin in serum samples was measured using an enzyme-linked immunosorbent assay (HyCult Technologies). After thawing, serum samples were diluted 20 times and assayed as recommended by the manufacturer. Detection range was 0.14–100 ng/L.

### Bacteria

Uropathogenic *E. coli* strain CFT073 was isolated from a patient with acute pyelonephritis and expresses type 1-, P- and S fimbriae as well as alpha-hemolysin. Bacteria were grown overnight on blood agar at 37°C, and then in LB broth for another 4 hours to reach logarithmic phase of growth. Bacteria were harvested by centrifugation and then washed twice with PBS. The bacterial concentration was measured by spectrophotometry and confirmed by viable count.

### Bladder biopsy infection

Bladder biopsies were immediately transferred to serum-free DMEM (Invitrogen) containing low-dose gentamicin (1 µg/mL) with or without *E. coli* CFT073 at 10^8^ CFU/mL and incubated at 37°C for 2 hours. Biopsies were then gently washed in PBS and fixed in 4% paraformaldehyde (PFA) or homogenized for RNA extraction using RNeasy Micro Kit (Qiagen) according to the instructions from the manufacturer. Fixed tissue was embedded in paraffin and sectioned at 4 µm.

### Cells

TERT-NHUC, normal human telomerase-immortalized urothelial cells were kindly provided by Prof M Knowles, University of Leeds. These cells resemble normal urinary bladder cells to a high degree [34]. Cells were maintained in Keratinocyte Serum-Free Medium supplemented with bovine pituitary extract and epidermal growth factor (Invitrogen). Normal human bladder epithelium cells (HBEP) were obtained from CELLnTEC and cultured in bladder epithelium medium (CnT-58, CELLnTEC). All experiments with normal bladder cells were performed within five passages. Urinary bladder cell lines T24 (ATCC No. HTB-4) and 5637 (ATCC No. HTB-9) and kidney epithelial cell line A498 (ATCC No. HTB-44) were maintained in McCoy's medium, RPMI-1640 (Invitrogen) and Eagle's MEM (Sigma-Aldrich) respectively, supplemented with 10% fetal bovine serum (FBS). Cell lines and HBEP were maintained in 5% CO_2_ and 80% humidity at 37°C and 35°C respectively.

### Cell experiments

Cell experiments were carried out in 6 or 24 well cell culture plates (Costar), or Falcon Primaria tissue culture plates (BD Falcon) for TERT-NHUC cells. When cells were near-confluent, medium containing 2% charcoal treated FBS for T24 and 5637, or fresh serum-free medium for TERT-NHUC cells and normal bladder cells, was added, supplemented with 1,25D_3_ (a kind gift from Dr. M. Uskokovic, BioXell), 25D_3_ (Calbiochem) or vector alone (ethanol). 25D_3_ concentrations of 10^−7^ M were needed for a consistent *CAMP* response in bladder cells whereas 1,25D_3_ concentrations of 10^−8^ M resulted in similar induction. All cell experiments with vitamin D were performed away from direct light.

### RT- PCR

RNA was extracted using RNeasy Mini Kit (Qiagen) according to instructions. Concentration and purity of RNA was determined spectrophotometerically at 260 and 280 nm. An aliquot of 0.5–1 µg of RNA was reversely transcribed using M-MuLV Reverse Transcriptase (Finnzymes). Real-time PCR was performed on a RotorGene 3000 (Corbett Research) with primers and probes from Applied Biosystems; *CAMP* Hs00189038_m1, *CYP24A1* Hs00167999_m1, *CYP27B1* Hs00168017_m1, *VDR* Hs01045844_m1. 18S rRNA was used as housekeeping gene. For relative mRNA expression, fold change in gene expression was calculated using the 2^−ΔΔC^_T_ method. Since *CYP27B1*, *VDR* and *CYP24A1* are known to be expressed in the kidney epithelium, the kidney cell line A498 was used as a positive control. mRNA expression in bladder cells and tissue were therefore normalized to the levels in A498.

### Immunofluorescence confocal microscopy

Cells on cover-slips (VWR) were grown in the presence of 25D_3_, 1,25D_3_ or vector for 48 hours. For infection, 10^7^ *E. coli* CFT073 per mL was added for 90 minutes. Cells were fixed in 4% PFA in PBS. Antibodies and blocking sera were diluted in 0.1% Triton X-100 in PBS. Unspecific antibody staining was blocked using 10% rabbit serum (Sigma-Aldrich) for 60 minutes, followed by FX Signal Enhancer (Invitrogen) for 30 minutes. Primary antibodies used were: anti-cathelicidin monoclonal antibody 3D11 (HyCult Technologies) 1∶200 for 60 min, this antibody recognizes both the pro-form (hCAP18) and the cleaved form (LL-37); anti-VDR monoclonal antibody MA1-710 (Affinity BioReagents) 1∶400 for 30 minutes, anti-CYP27B1 polyclonal antibody D-20 (Santa Cruz) 1∶200 for 30 minutes and anti-CYP24A1 polyclonal antibody (Sigman Aldrich) 1∶150 for 30 minutes. Secondary antibodies were: AlexaFluor 594-conjugated rabbit anti-mouse, anti-rat or anti-goat antibody (Invitrogen) 1∶400 for 30 minutes, or FITC-conjugated anti-goat antibody (AbD Serotec) 1∶600 for 30 minutes. Nuclei were counterstained with DAPI (Invitrogen). Specificity of immunolabelling was controlled in preparations without primary antibody. Deparaffinized and rehydrated bladder sections were stained as above, except primary antibody staining was carried out at 4°C overnight. ProLong Gold antifade mounting medium (Invitrogen) was used. Images were acquired with a Leica TCS SP5 confocal microscope. Fluorescence intensity was quantified using the ImageJ image processing software (U. S. National Institutes of Health) [35]. For Live/Dead staining, cells were infected with 10^7^ *E. coli* CFT073 per mL for 60 minutes, washed to remove unbound bacteria and incubated for an additional 60 minutes. Cells were permeabilized with 0.2% saponin in PBS for 5 minutes, before adding Live/Dead Bacterial Stain (Invitrogen) for 15 minutes. Cells were then mounted and analyzed by microscopy immediately. For colocalization studies, GFP-labelled CFT073 strain LT004 (a kind gift from Prof Agneta Richter-Dahlfors, Karolinska Institutet) was used [36].

### Flow cytometry

Cells were exposed to 25D_3_, 1,25D_3_ or vector as described. After detachment by trypsinization, they were washed in PBS, fixed and permeabilized using the BD Cytofix/Cytoperm™ Plus according to instructions and stained with antibodies for 30–40 minutes at 4°C. Cells were stained with an anti-cathelicidin monoclonal antibody 1-1C12 1∶100 (HyCult Technologies) followed by an AlexaFluor 488-conjugated anti-mouse IgG secondary antibody (Invitrogen). The cells were analyzed on a Becton Dickinson FACScalibur™ flow cytometer using the CellQuest™ software (BD Biosciences Immunocytometry systems).

### Antibacterial assay

After stimulation of bladder cells with 10^−8^ M 1,25D_3_ or vector for 48 hours, 150 µL supernatant was incubated with 50 µl of 10^4^ CFU/mL *E. coli* CFT073 on a shaker at 37°C for 90 minutes. An aliquot was plated on blood agar and colonies were counted after incubation overnight. In selected experiments, 1 µg/mL anti-cathelicidin monoclonal antibody 3D11 (HyCult Technologies) or IgG_1_ isotype control (BD Biosciences) was added to the cell supernatant for 120 minutes prior to bacterial coincubation. For intracellular antibacterial assay, vitamin D-stimulated cells were lysed in 1% Triton X-100 in PBS, centrifuged at 8000×g for 5 minutes and used for antibacterial assay as described above.

### Statistics

All statistical analyses were performed using GraphPad Prism 4.01. Mann-Whitney test, Wilcoxon rank test or ANOVA with appropriate post test were used. Spearman rank test was used for correlation between 25D_3_ and cathelicidin levels in serum. *P*-level of less than 0.05 was considered significant.
